# Prevalence of delirium, depression, anxiety, and post-traumatic stress disorder among COVID-19 patients: protocol for a living systematic review

**DOI:** 10.1186/s13643-020-01507-2

**Published:** 2020-11-06

**Authors:** Jiyuan Shi, Ya Gao, Liang Zhao, Yuanyuan Li, Meili Yan, Ming Ming Niu, Yamin Chen, Ziwei Song, Ruixing Zhang, Lili Zhang, Jinhui Tian

**Affiliations:** 1 Evidence-Based Medicine Center, Lanzhou University, No.199, Donggang West Road, Lanzhou City, 730000 Gansu Province China; 2 School of Nursing, Zhengzhou University, No. 100, Science Avenue, Zhengzhou, 450000 Henan Province China; 3 School of Nursing, Southern Medical University, No. 1023-1063, South Shatai Road, Baiyun District, Guangzhou, 510000 Guangdong Province China

**Keywords:** Living systematic review (LSR), COVID-19, Infection, Mental health

## Abstract

**Background:**

Previous studies on the impact of corona virus disease 2019 (COVID-19) on the mental health of the patients has been limited by the lack of relevant data. With the rapid and sustained growth of the publications on COVID-19 research, we will perform a living systematic review (LSR) to provide comprehensive and continuously updated data to explore the prevalence of delirium, depression, anxiety, and post-traumatic stress disorder (PTSD) among COVID-19 patients.

**Methods:**

We will perform a comprehensive search of the following databases: Cochrane Library, PubMed, Web of Science, EMBASE, and Chinese Biomedicine Literature to identify relevant studies. We will include peer-reviewed cross-sectional studies published in English and Chinese. Two reviewers will independently assess the methodological quality of included studies using the Joanna Briggs Institute Prevalence Critical Appraisal tool and perform data extraction. In the absence of clinical heterogeneity, the prevalence estimates with a 95% confidence interval (CI) of *delirium*, depression, anxiety, and post-traumatic stress disorder (PTSD) will be calculated by using random-effects model to minimize the effect of between-study heterogeneity separately. The literature searches will be updated every 3 months. We will perform meta-analysis if any new eligible studies or data are obtained. We will resubmit an updated review when there were relevant changes in the results, i.e., when outcomes became statistically significant (or not statistically significant anymore) or when heterogeneity became substantial (or not substantial anymore).

**Discussion:**

This LSR will provide an in-depth and up-to-date summary of whether the common neuropsychiatric conditions observed in patients hospitalized for severe acute respiratory syndrome (SARS-CoV) and Middle East respiratory syndrome (MERS) are also prevalent in a different stage of COVID-19 patients.

**Systematic review registration:**

PROSPERO CRD42020196610

## Background

The global outbreak of the coronavirus disease 2019 (COVID-19) has been designated as a pandemic that has directly affected over 32 million people, with more than nine hundred and ninety thousand fatalities [1, 2]. Previous research focusing on pandemics confirmed that individuals who had experienced public health emergencies reported varying degrees of psychological disorders even after the event ended or they were cured and discharged from the hospital [3–6]. Patients with confirmed and suspected infections may suffer from repeated psychiatric (disorders, symptoms, and signs listed in category 06 (mental, behavioral, or neurodevelopmental disorders) of the 11th edition of the ICD) and neuropsychiatric (psychiatric disorders, symptoms, and signs that are the result of brain damage or disease) due to multiple reasons [3], such as progression of the disease, adverse drug reaction, social isolation, uncertainty, and physical discomfort [7–9].

A recently published systematic review and meta-analysis indicated the prevalence of delirium as a common occurrence among patients hospitalized due to severe coronavirus infections (severe acute respiratory syndrome (SARS-CoV) and Middle East respiratory syndrome (MERS)), whereas post-traumatic stress disorder (PTSD), anxiety, depression, and fatigue were observed in the subsequent months [3]. There exists some preliminary/unpublished data showing psychiatric and neuropsychiatric presentations in COVID-19 patients [3]. Since the spread of COVID-19, there has been extensive research on the topic globally, translating into an unprecedented number of publications, approximately 59 articles per day, probably higher than observed for any other disease [10]. It is essential to collect continuously updated data to provide convincing evidence for patients, healthcare workers, and policymakers. A living systematic review (LSR) retains the benefits of a systematic review and accepts continual updating of the relevant data without compromising the methodological rigor [11–14].

The aim of this study is to provide a living systematic review for synthesizing rapid and continual updating of data on whether the common neuropsychiatric conditions observed in patients hospitalized for severe SARS-CoV or MERS-CoV are also prevalent in a different stage of COVID-19 patients. First, we will analyze the prevalence of delirium in patients diagnosed with COVID-19. Second, we will analyze the prevalence of depression, anxiety, and post-traumatic stress disorder in patients diagnosed with COVID-19.

## Methods/design

### Study design

This systematic review has been reported in accordance with the Preferred Reporting Items for Systematic Reviews and Meta-Analyses Protocols (PRISMA-P) statement and has been registered on PROSPERO (CRD42020196610) [15].

### Eligibility criteria

#### Participants (population)

We will include studies involving the adult population (≥ 18 years of age) diagnosed (laboratory-confirmed infection) with COVID-19. We will exclude studies involving populations with other coronavirus diseases (SARS or MERS unless the trial authors provided subgroup data for people with COVID-19).

#### Condition or outcome(s) of interest

We will include studies involving patients who are diagnosed with four types of psychiatric and neuropsychiatric syndromes (delirium, anxiety (e.g., generalized anxiety, and panic attack), depression, or post-traumatic stress disorder), with no age, gender or setting, location, or ethnicity restrictions [16]. The psychiatric and neuropsychiatric syndromes should be diagnosed by a trained researcher or health professional according to the criterion defined by ICD-10, ICD-11, DSM-IV, DSM-V, and Chinese Classification of Mental Disorders-3 (CCMD-3) or by validated psychometric scales (e.g., clinician-administered PTSD scale for PTSD) with established cutoffs approved by psychologists (RXZ). We will exclude studies that explored the indirect effects of SARS-CoV-2 on the mental health of family members, care providers, or isolated people who did not infect and studies that did not report symptom measurement methods or diagnostic criteria. We will also exclude patients with a prevalence of psychiatric and neuropsychiatric syndromes reported before being diagnosed with COVID-19.

#### Type of outcome

The primary outcomes that will be analyzed are the prevalence of delirium (acute phase of illness) and the prevalence of anxiety (e.g., generalized anxiety, panic attack), depression, and post-traumatic stress disorder (post-illness phase).

#### Study design and context

Cross-sectional studies will be the most appropriate study design to answer the question of prevalence; therefore, we will include only peer-reviewed cross-sectional studies published in English and Chinese. Conference abstracts, commentaries, or opinion pieces will also be excluded because they lack adequate information for meta-analysis. If more than one dataset was reported for the same group of patients, we will use the results of the longest post-follow-up that were assessed. Moreover, if a study has multiple time points in the same studies, we will use the most recent time point for the prevalence report.

### Outcomes and prioritization

Previous studies confirm that delirium occurs commonly in hospitalized patients with virus infection (acute phase of illness) while depression, anxiety, and post-traumatic stress disorder occur in subsequent months (post-illness phase). However, data on the acute effects of the COVID-19 are limited and no data exist on the post-illness phase. Therefore, the primary outcomes that will be analyzed are the prevalence of delirium (acute phase of illness) and the prevalence of anxiety (e.g., generalized anxiety, panic attack), depression, and post-traumatic stress disorder (post-illness phase). The outcome will be classified as examining the acute or post-illness psychiatric consequences of infection on the basis of whether the information is collected during the patient’s illness or the period after the illness.

### Search strategy

A senior investigator (Y.G.) will examine the published and gray literature sources to extract the studies reporting the prevalence of depression, PTSD, anxiety, or delirium in COVID-19 patients. An experienced medical information specialist (J.H.T.) will further check and approve the search methodology. We will conduct a comprehensive search of the Cochrane Library, PubMed, Web of Science, EMBASE, and Chinese Biomedicine Literature to extract articles/abstracts published between the inception of this disease (1 December 2019) until the completion of this review will be included. There will be no restrictions on language or year of publication. We will also thoroughly search the reference lists of the relevant reviews and research trials. We have presented the search strategy using PubMed as an example in Table 1. The search strategy will be adapted to fit other online databases as well.

**Table 1:** Search strategy of PubMed database

#1 “Stress Disorders, Post-Traumatic”[Mesh] OR “Post Traumatic Stress Disorder*”[Title/Abstract] OR “Post traumatic Neuroses”[Title/Abstract] OR “Post traumatic Stress Disorder*”[Title/Abstract] OR “Post Traumatic Neuroses”[Title/Abstract] OR “Post-Traumatic Neuroses”[Title/Abstract] OR “Post-Traumatic Stress Disorder*”[Title/Abstract] OR “Post Traumatic Stress Disorder*”[Title/Abstract] OR “Moral Injur*”[Title/Abstract] OR “Delayed Onset Post-Traumatic Stress Disorder”[Title/Abstract] OR “Delayed Onset Post Traumatic Stress Disorder”[Title/Abstract] OR “Chronic Post-Traumatic Stress Disorder*”[Title/Abstract] OR “Chronic Post Traumatic Stress Disorder*”[Title/Abstract]	
#2 “Depressive Disorder”[Mesh] OR “Depression”[Mesh] OR “Depression*”[Title/Abstract] OR “Depressive Symptom*”[Title/Abstract] OR “Emotional Depression*”[Title/Abstract] OR “Depressive Disorder*”[Title/Abstract] OR “Depressive Neuroses”[Title/Abstract] OR “Depressive Neurosis”[Title/Abstract] OR “Endogenous Depression*”[Title/Abstract] OR “Depressive Syndrome*”[Title/Abstract] OR “Neurotic Depression*”[Title/Abstract] OR “Melancholia*”[Title/Abstract] OR “Unipolar Depression*”[Title/Abstract]	
#3 “Delirium”[Mesh] OR “Delirium”[Title/Abstract] OR “Subacute Delirium*”[Title/Abstract] OR “Delirium of Mixed Origin”[Title/Abstract] OR “Mixed Origin Delirium*”[Title/Abstract]	
#4 “Anxiety”[Mesh] OR “Anxiet*”[Title/Abstract] OR “Hypervigilance”[Title/Abstract] OR “Nervousness”[Title/Abstract] OR “Social Anxiet*”[Title/Abstract]	
#5 “COVID-19” [Supplementary Concept]	
#6 “2019 novel coronavirus disease”[Title/Abstract] OR “COVID19”[Title/Abstract] OR “COVID-19 pandemic”[Title/Abstract] OR “SARS-CoV-2 infection”[Title/Abstract] OR “COVID-19 virus disease”[Title/Abstract] OR “2019 novel coronavirus infection”[Title/Abstract] OR “2019-nCoV infection”[Title/Abstract] OR “coronavirus disease 2019”[Title/Abstract] OR “coronavirus disease-19”[Title/Abstract] OR “2019-nCoV disease”[Title/Abstract] OR “COVID-19 virus infection”[Title/Abstract]	
#7 #1 OR #2 OR #3 OR #4	
#8 #5 OR #6	
#9 #7 AND #8	

### Update plan

We will perform identical search operations at regular pre-defined intervals to identify newly published data. There are no robust standards for the update frequency based on current research; however, due to the unprecedented number of publications on COVID-19, the literature searches will be updated every 3 months. We will perform meta-analysis if any new eligible studies or data are obtained. We will resubmit an updated review when there were relevant changes in the results, i.e., when outcomes became statistically significant (or not statistically significant anymore) or when heterogeneity became substantial (or not substantial anymore) [11, 12]. We chose this updating frequency to allow quick updates and to highlight the most recent information to the researchers, clinicians, nurses, and policymakers [11, 14, 17]. Considering the publication process of systematic reviews is usually several months from submission of the manuscript to acceptance/publication, we will choose the format of pre-prints during the process of peer review for timely publication.

### Selection process

Original literature search records will be imported into the Endnote X9 software tool (Thomson Reuters, New York, NY, USA) management software. Two independent reviewers (J.Y.S, L.Z.) screened out possibly relevant studies by titles and abstracts extraction sheet to exclude records that did not meet the inclusion criteria. Then, the same two reviewers screen out the studies that met the inclusion criteria by evaluating the full text. The selection process will be conducted under the supervision of a psychologist (RXZ). Any disagreement will be resolved by the reviewer (JHT). We will contact the corresponding or other primary authors to obtain missing data or insufficiently reported data after selecting the studies. In addition, we will estimate missing data if they can be extracted from tables or figures. Studies with missing data that cannot be obtained will be excluded for reasons.

### Data collection process

All reviewers (JHT, JYS, ZL, and MMN) involved in this study will previously pilot the form on a random sample of three included studies to ensure the agreement among the interpretation of data items. One reviewer (LZ and MMN) extracts data from the included studies using a data extraction sheet, and a second reviewer (JHT and JYS) will verify the extracted data. The data extraction will be performed on Microsoft Excel 2016.

### Data items

We will extract population details (e.g., age, ethnicity, and gender); study settings (e.g., country, study site); sample size; study design (e.g., cohort studies); diagnostic criteria for the COVID-19 infection (such as WHO criteria); criteria for the definition of delirium, depression, anxiety, and PTSD (e.g., ICD-11); the criteria for the definition of delirium, depression, anxiety, and PTSD (e.g., clinician-administered PTSD scale for PTSD); timing (acute or post-illness phase); and follow-up time.

### Critical appraisal

The critical appraisal for SRs of prevalence should consider the diversity of review types; the critical appraisal checklist developed for systematic reviews of prevalence was deemed appropriate to use when assessing studies included in systematic reviews of prevalence data [18, 19]. Two independent reviewers (JYS and YG) will assess the methodological quality of included studies using the Joanna Briggs Institute Prevalence Critical Appraisal tool [19]. We will classify each individual study as having a low, high, or unclear risk of bias as described in Table 2. Any disagreement regarding the inclusion of studies will be resolved by discussion and adjudicated by the third reviewer (J.H.T.).

**Table 2:** Risk of bias assessment for included studies

Study design	Tool	Domains/checklist	Overall risk of bias judgment
Cross-sectional studies	JBI Prevalence Critical Appraisal Tool	1. Was the sample frame appropriate to address the target population? 2. Were study participants sampled in an appropriate way? 3. Was the sample size adequate? 4. Were the study subjects and the setting described in detail? 5. Was the data analysis conducted with sufficient coverage of the identified sample? 6. Were valid methods used for the identification of the condition? 7. Was the condition measure in a standard, reliable way for all participants? 8. Was there appropriate statistical analysis? 9. Was the response rate adequate, and if not, was the low response rate managed appropriately?	An item would be scored “0” if it was answered “No” or “Unclear”; if it was answered “Yes”, then the item scored “1”. Methodological quality will be considered “low,” “moderate,” and “high” if three or less, four to six, and seven to nine criteria will be met, respectively.

*JBI* Joanna Briggs Institute

### Protocol deviations

Any significant deviations between the protocol and the final review will be reported with reasons and describe what impact these changes have on the results, such as the planned subgroup analysis which cannot be analyzed due to insufficient data.

### Data analysis

The Stata (v13.0; StataCorp) will be used for statistical analysis. In the absence of clinical heterogeneity (determined by RXZ and JHT), the pooled prevalence with a 95% confidence interval (CI) of delirium, depression, anxiety, and PTSD will be calculated by using random-effects model to minimize the effect of between-study heterogeneity separately. The statistical heterogeneity will be examined using *I*^2^ statistic, with an *I*^2^ of more than 75% indicating substantial heterogeneity [20], and the Cochran’s Q and the Tau^2^ will also be reported with a *P* value of < 0.05 considered statistically significant (heterogeneity). The publication bias will be examined using the Egger test or the symmetry of the funnel plot. In the Egger test, bias will be significant when *p* value < 0.05.

### Subgroup analysis

If sufficient data are available, the following subgroup analyses will be planned for main outcomes if data are sufficient: age (< 60 vs. ≥ 60 years), World Health Organization (WHO) region (e.g., Americas region vs. European region), setting (developed countries vs. developing countries defined by United Nations (UN) Millennium Development Goals (MDGs) regions), gender (male vs. female), diagnostic criteria (e.g., DSM-IV/V or ICD-10/11 vs CCMD-3), and follow-up time.

### Sensitivity analyses

We will perform sensitivity analyses to assess the influence of the study’s methodological quality. To do this, we will repeat any meta-analyses by excluding data from studies classified as low quality.

### Presenting and reporting results

We will present the step-by-step process of study selection methods in the form of a flow diagram. The characteristics and quality assessment of the included studies will be presented in tables. Forest plots will be generated displaying pooled estimates with the corresponding 95% CI.

## Discussion

Coronaviruses have resulted in two severe outbreaks of the SARS, however, before SARS-CoV-2. Previous coronaviruses have been associated with delirium signs in the acute stage and fatigue, depression, PTSD, and anxiety in the post-illness stage [3]. However, the lack of adequate data on COVID-19 patients limited the previous study to investigate and conclude the effects of the SARS-CoV-2 infection on patients’ mental health. Given that the rapid and sustained growth of publication of COVID-19 research, we will perform an LSR to comprehensive and continuous synthesis updated data to explore the prevalence of delirium, depression, anxiety, and PTSD in COVID-19 patients. They anticipate potential challenges of our planned SRs methods is that we will conduct a comprehensive literature search of four English databases and one Chinese database; however, excluding non-Chinese and non-English studies may cause a publication bias. Therefore, we will comprehensively retrieve the reference lists of the related SRs to obtain more additional studies.

## Data Availability

Not applicable.

## Abbreviations

LSR: Living systematic review
PTSD: Post-traumatic stress disorder
SARS: Severe acute respiratory syndrome
MERS: Middle East respiratory syndrome
CCMD-3: Chinese Classification of Mental Disorders-3
WHO: World Health Organization
UN: United Nations
MDGs: Millennium Development Goals
